# Effects of Green Tea Powder, Pomegranate Peel Powder, Epicatechin and Punicalagin Additives on Antimicrobial, Antioxidant Potential and Quality Properties of Raw Meatballs

**DOI:** 10.3390/molecules26134052

**Published:** 2021-07-02

**Authors:** Tuğba Demir

**Affiliations:** Department of Food Hygiene and Technology, Faculty of Veterinary Medicine, Sivas Cumhuriyet University, Sivas 58040, Turkey; tugba@cumhuriyet.edu.tr; Tel.: +90-(346)-219-1010-3618; Fax: +90-(346)-219-1812

**Keywords:** phenolic compounds, meatball, antimicrobial activity, food preservation, food-borne pathogens

## Abstract

Alternative technologies, which have been developed in order to meet the consumers’ demand for nourishing and healthy meat and meat products, are followed by the food industry. In the present study, it was determined, using the HPLC method, that green tea contains a high level of epicatechin (EP) under optimal conditions and that pomegranate peel contains a high level of punicalagin (PN). Green tea, pomegranate peel, EP and PN were added to meatballs at different concentrations in eight groups. The antioxidant capacities of extracts were measured. The antimicrobial activity was examined for 72 h using three different food pathogens. The highest level of antimicrobial activity was achieved in the 1% punicalagin group, whereas the minimum inhibition concentration (*L. monocytogenes*, *S. typhimurium*) was found to be 1.87 mg/mL. A statistically significant decrease was found in FFA, POV and TBARS levels of meatballs on different days of storage (*p* < 0.05). When compared to the control group, the bioactive compounds preserved the microbiological and chemical properties of meatballs during storage at +4 °C (14 days). It was concluded that the extracts with high EP and PN concentrations can be used as bio-preservative agents for meat and meat products.

## 1. Introduction

Food-borne diseases are among the major public health issues throughout the world and they can cause severe complications. International medical authorities draw attention to the importance of food processing and food preservation [1,2]. Advancing technology and increasing R&D works enable the meat and meat product industry to improve themselves and to gain new achievements. The consumers expect meat and meat products to be healthy and safe, as well as being functional and nourishing.

Natural compounds such as fatty acids, minerals, vitamins, natural antioxidants, dietary fibers, probiotics and bioactive peptides are among the technologies used in developing qualified meat products [3,4,5]. Meat and meat products are ideal substrates for development and reproduction of common food-borne pathogens. It makes these foods highly risky and easily spoiled, causing potential public health issues [6,7].

Meat and meat products that are not stored under appropriate conditions are accepted to be the most important sources of food-borne pandemics arising from pathogens such as *Salmonella enteritidis*, *Escherichia coli* O157: H7, *Bacillus cereus*, *Bacillus subtilis*, *Listeria monocytogenes* and *Staphylococcus aureus* [8]. Beside the risk of microbial contamination, lipid and protein oxidations during the storage period are among the risk parameters [9].

Oxidative changes (lipid and protein oxidation) occur faster in minced meat. It was reported that it might be because the meat surface area in contact with air significantly increases after mincing the meat [10].

Oxidative reactions are among the main mechanisms of meat spoilage after the butchering process. The high levels of myoglobin and ferrous content of bovine meat, which is in the class “red meat”, are one of the reasons for strong peroxide formation [11].

Increasing the functional and nutritional value of foods and prolonging the storage period by adding phenolic compounds into meats and meat products are among the focus points of studies carried out on this subject [12]. Natural additives containing phenolic compounds are added directly into meats and meat products in order to improve the antimicrobial and antioxidant properties of the product, as well as the quality characteristics during the storage period [13]. Many studies were carried out by using extracted forms and volatile oils of plants [14]. Appropriate application of plant-based extracts in meat products depends on the type of process, form of extraction and type of meat [15]. Different results can be achieved by using the same natural additive. Beside protecting meat and meat products from microbial and oxidative damages, natural extracts having a high level of phenolic content also offer many metabolic benefits to human health [16].

Because of the irregular use of antibiotics in recent years and the medication-resistant development among human pathogen bacteria, the interest in phenolic compounds having antioxidant and antimicrobial action increased. It was reported, in different studies, that these compounds inhibit the development of various bacteria and molds in these environments, they may prevent various infectious diseases that may arise from these microorganisms and they may be useful in controlling pathogens [17].

Phenolic compounds isolated from plants inhibit different enzymes and have positive effects on human health. The anti-allergic, anti-glycemic, anti-cholesterol, anti-inflammatory, antithrombic, vasodilator and tranquilizer properties of herbal extracts were reported in previous studies [18]. Moreover, it was also reported that coronary heart disease risk decreases among individuals consuming foods with a high level of phenolic content [19].

There are studies carried out by adding green tea extract into different meats (other than red meat) and meat products and investigating the microbial and biochemical properties [20,21]. In one of these studies, it was reported that green tea additives protected sausages from oxidative changes [22]. In another study, it was reported that the Frankfurt-type sausages enriched with green tea extract had better storage and quality characteristics when compared to the control group [23]. 

Various in vitro and in vivo studies confirmed that pomegranate is rich in phenolic compounds [24,25]. It was also found, in previous studies, that the peel of pomegranate has a higher level of phenolic compounds [26]. There are studies examining the use of pomegranate peel as a bio-protective agent for meat and meat products. It was determined that the functional and nutritional properties were improved, the products showed a higher level of antioxidant activity [27], they had an antimicrobial effect on microorganisms and the quality characteristics were enhanced by controlling lipid and protein oxidation [28].

Adding green tea and pomegranate extracts obtained under optimal conditions and EP and PN phenolic compounds into meatballs, the present study aims to prolong the storage period (+4 °C) and to minimize the levels of microbiologic and oxidative reactions that may develop during storage.

The first objective is to determine the phenolic compositions of green tea and pomegranate peel extracts, which were obtained under optimum conditions, by using the HPLC method and to reveal the highest phenolic content among these two extracts. The other objective is to prolong the storage period (+4 °C) and to minimize the levels of microbiologic and oxidative reactions by adding selected extracts (green tea and pomegranate peel) and high phenolic standards (EP and PN) into meatballs.

## 2. Material and Methods

### 2.1. Preparation of Microbial Suspensions and Media

Three pathogens strains (*E. coli* ATCC 25922, *L. monocytogenes* ATCC 7644 and *S. typhimurium* ATCC 14028) were used in this study. All ATCC strains and media were purchased from Sigma-Aldrich (St. Louis, MO, USA). Before use, all pathogens were incubated (at 37 °C for 24 h) in Tyriptic soy broth. Tubes were prepared for each bacterial strain in sterile deionized water, with a turbidity of 1 McFarland, to perform MIC determination using a McFarland standard. Serial dilutions between 10^−1^ and 10^−5^ of each bacterial suspension were streaked on TSA petri dishes in order to count the microorganisms and verify that the number of bacteria in the samples was appropriate for the performance of the tests. Mueller Hinton Broth (MHB), used for performing the determination of MIC, was enriched with a suitable volume, obtaining 0.5% (*v*/*v*) solutions identified.

### 2.2. Preparation of Extracts, Standard Phytochemicals and Meatball Groups

Green tea powder (GTP) and pomegranate peel powder (PPP) were obtained from Sivas local markets. In the laboratory environment, the oven was kept at 40 °C for two hours for the first sterilization. Extraction solution was prepared before grouping. Powdered samples (1:5 g/mL water) were extracted for 24 h using a Soxhlet extractor. Six different conditions were applied on the amount of solvent and extraction time for the extraction of powder samples [29]. The working condition with the highest phenolic and flavonoid content was chosen. Conditions: 1:2.5 g/mL, 1:5 g/mL, 1:10 g/mL water, 12 h and 24 h, respectively. The extract was concentrated to dryness under low pressure and controlled temperature (40–50 °C).

HPLC grade EP and PN were used compared against the natural extracts. In the preparation of standard solutions, the purchased firm was made by examining the SDS package insert to Sigma-Aldrich (St. Louis, MO, USA). The control (G0) and eight treatment groups were determined for meatballs. These groups were 0.5% green tea (G1), 1% green tea (G2), 0.5% EP (G3), 1% EP (G4), 0.5% pomegranate peel (T1), 1% pomegranate peel (T2), 0.5% PN (T3) and 1% PN (T4), respectively.

### 2.3. Determination of Total Phenolic and Flavonoid Compounds

The total phenolic content of the extract was determined according to the method described using the Folin–Ciocalteu phenol reagent [30]. A total of 2 N 100 μL was mixed with extracts/standard gallic acid solutions (100 μL extract/100 μL of standard), 2.3 mL of purified water and 1 mL of aqueous sodium carbonate solution (7%). A standard curve was created with gallic acid. Then (25 °C, 2 h), the absorbance at 750 nm wavelength was measured by the spectrophotometer. The total amounts of flavonoids of the extracts were determined according to the method provided by Zhishen et al. (1999) [31]. The extracts were made to react with 5% NaNO_2_, 10% AlCl_3_ and NaOH (1 M), respectively. Quercetin was used as a standard to determine the total flavonoid content of the extracts. A standard curve was created with quercetin and the absorbance was measured at 510 nm. Total phenolic compounds were expressed as gallic acid equivalent (GAE) and total flavonoid compounds as quercetin equivalent (QE). All analyses were performed in triplicate.

### 2.4. Determination of Phenolic Composition with HPLC 

The GTP and PPP compositions were determined by HPLC on an Agilent 1200 chromatograph (Agilent Technologies, Santa Clara, CA, USA). The work by Aloqbi et al. [32] was used, by modifying the HPLC method. Water (solvent A) and acetonitrile (solvent B) were used as the mobile phase. The dilution was performed on a Zorbax SB-C18 chromatographic column (150 mm × 4.6 mm × 3.5 μm) (Agilent Technologies Santa Clara, CA, USA). Other specifications were: flow rate of carrier gas (helium) through column, 0.5 mL/min, pomp temperature, 30 °C, and injection volume, 3 μL. The gradient elution mode was 0–8 min 100% A, 5 min 100–88% A, 10 min 88–68% A and 7 min 68–55% A. Detection was performed using a diode-matrix detector with a signal recording at a wavelength range of 0–250 nm.

### 2.5. Preparation of Meatballs

Ground beef was purchased from a local butcher in Sivas, Turkey. Beef minced meat and salt were used in preparing the samples. GTP, PPP, EP or PN were added to the formulation of meatballs. The remaining group without any addition was used as control. Kneading was performed after each component was added to the meatball mortar at the rate of 0.5% and 1%, respectively. In order for the meatballs to be of standard size and shape, samples were prepared as one meatball weighing 25 g, with the help of a shaping machine. All the experiments were performed three times (three different groups).

### 2.6. Determination of Minimum Inhibitory Concentration (MIC)

MIC was used as a measure of antimicrobial performance of phytochemicals. Determination of MIC assay was performed as described by Weerakkody et al. [33], with some modifications to the method. Aliquots of 100 mL of each bacterial suspension were prepared, with a turbidity of 1 McFarland, corresponding approximately to 10^7^ CFU/mL, and 10 mL were added to each tube containing the serial dilutions (from 30 to 0.46 mg/mL) of the phytochemicals (GTP, PPP, EP and PN). The positive control was obtained by preparing a test tube containing 2 mL of Mueller Hinton Broth (MHB) and 100 mL of the bacterial suspension; the negative control contained 2 mL of MHB, 100 mL of the bacterial suspension and 10 mL of the antibiotic ampicillin. All tubes were tightly capped to prevent evaporation. The experiment started at time “0”; then, at “1st, 24th and 72th” h, each tube concentration with color change was determined as MIC. Subsequently, the determined concentration was sowed and the MBC (minimum bactericidal concentration) was confirmed. A volume of 100 mL of the suspension was spread on TSA Petri dishes, which were incubated at 37 °C aerobically; after 24 h, the colonies were counted. The tubes were then incubated at 37 °C aerobically. The effect of phytochemicals on bacteria was monitored at intervals of 24 h, up to 72 h, by seeding 100 mL of the suspensions on TSA petri dishes, as previously described.

### 2.7. Biochemical Analysis and Microbial Assessment

#### 2.7.1. Antioxidant Capacity and Equivalence Calculations

The GTP, PPP extracts and EP and PN were evaluated for antioxidant capacity using the colorimetric Folin–Ciocalteu, 2,2-diphenyl-1-picrylhydrazyl radical (DPPH) and ferric reducing antioxidant power (FRAP) assays, 2,2′-azinobis-(3-ethylbenzothiazoline-6-sulfonate) (ABTS) [30]. Analyses were performed in triplicate and results obtained from using standard calibration curves and expressed as mmol Trolox equivalents/g [34,35].

#### 2.7.2. Free Fatty Acid Values (FFA)

Samples (5 g) were dissolved with 30 mL of chloroform using a homogenizer (IKA Ultra-Turrax T18 Basic, Staufen, Germany) (10,000 rpm; 1 min). Whatman No. 1 was used to remove the filtrate. The resulting filtrate (added phenolphthalein) was titrated (0.01 N KOH). The FFA value was calculated with the formula [36].

#### 2.7.3. Peroxide Value (POV)

The POVs of meatball samples were determined according to the method by Volpe et al. [37] and Shahbazi et al. [38]. The samples (3 g) were weighed and heated in a water bath at 60 °C for 3 min (to melt the oil). The flasks were then shaken for 3 min with acetic acid-chloroform solution (3:2 by volume). Whatman No. 1 was used to remove the filter. Saturated potassium iodide solution (0.5 mL) was added to the final filtrate (indicator starch). The titration process was continued against the standard sodium thiosulfate solution. The POV was determined in the total lipid extracts and calculated with the formula. Results are given as POV (meq/kg). 

#### 2.7.4. Thiobarbituric Acid Reactive Substances (TBARS)

Lipid oxidation was measured with 2-thiobarbituric acid reagent, as modified by Sharma et al. [39]. Values (1,1,3,3-tetraethoxypropane) were calculated by drawing the standard curve (y = ax + b) and expressed as milligram of malondialdehyde per kilogram of meatball sample (mg MA/kg).

### 2.8. Statistical Analysis

All the tested sample data are reported as the mean and standard deviation. One way analysis of variance (ANOVA) with the SPSS statistical software (SPSS version 19.0 software, SPSS; Chicago, IL, USA) was used to determine the significant differences. All the mean values were used for the Duncan’s multiple test to perform post-hoc verification (*p* < 0.05).

## 3. Results and Discussion

### 3.1. Total Phenolic Content and Antioxidant Activity and Biochemical Analysis (FFA, POV and TBARS)

In the present study, GTP and PPP were extracted under optimum extraction conditions and the phenolic compositions were determined. It was found that GTP extract contains a high level of EP (22.18 ± 0.64 mg/g) and PPP extract contains a high level of PN (34.32 ± 1.37 mg/g).

**Figure 1:**
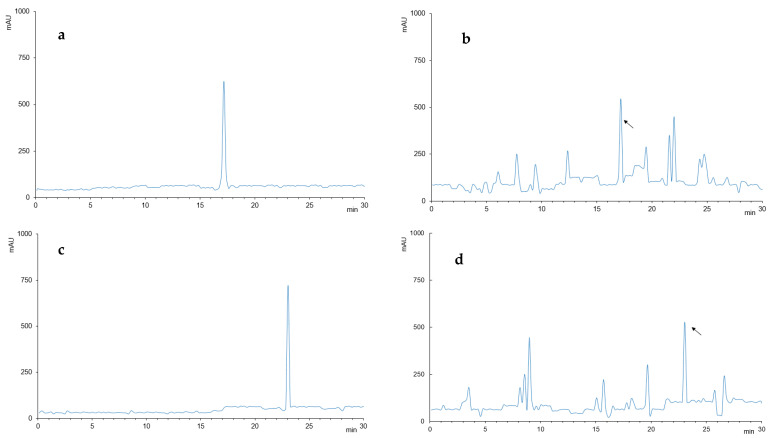
HPLC analysis of GTP, PPP, EP and PN. (**a**) EP, standard; (**b**) GTP, 1:5 mg/mL in water; (**c**) PN, standard; (**d**) PPP, 1:5 mg/mL in water.

The HPLC analysis results are shown in Figure 1. The EP peaks of aqueous GTP extract are shown together with standards in Figure 1b, whereas the PN peaks of PPP extract are shown together with standards in Figure 1d. Thus, the present study was designed by considering that GTP has a high level of EP content and PPP has a high level of PN content.

High fat concentration in the meat does not support the contact between phenolic compounds, which accumulate during the hydrophilic phase of meat and pathogen microorganisms [40]. Thus, it is thought that the pathogens developing during the storage of meat cannot be taken under control while using volatile oils, which contain phenolic compounds, for improving the quality characteristics of meat and meat products. It was reported that the synergetic and antagonistic effects of extracts (instead of essential oils) obtained using different solvents contribute to the antimicrobial activity of phenolic compounds [41]. The selection of herbal extracts (GTP, PPP) instead of essential oils and determining the antimicrobial activity of selected extracts are supported by the results reported by Tajkarimi et al. [41].

Nowadays, consumers’ concerns about meat and meat products increase gradually, because they have information about the oxidation, spoilage and food-borne pathogens that may develop in meat and meat products. Among the protection methods reported in the literature, the bio-additives made of phenolic compounds are considered to be among the popular methods. Considered as natural additives, phenolic compounds are beneficial for health and minimize the spoilage capacity of food. Containing phenolic compounds with high antioxidant capacities, the DPPH, ABTS and FRAP values of GTP and PPP extracts are presented in Table 1.

Total phenolic content and antioxidant activity of GTP, EP, PPP and PN extracts are shown in Table 1. Among the antioxidant capacity values measured, the lowest value was found in GTP extract (11.44 ± 0.17, 4.91 ± 0.54 and 19.07 ± 0.24 mmol TE/g). Although they had lower values when compared to the analyzed groups, the results were significant and in parallel with antioxidant activity results of GTP extract reported in the literature. The catechin and catechin-derivative contents of green tea are strong antioxidants preventing free radical formation and risk of future diseases [42]. Adding PPP and PP having a high level of phenolic content and high levels of free radical binding and antimicrobial activity into different meat products, bio-protective properties were obtained during the storage period [43,44]. In the present study, it was found that the values recorded were within the limits reported in the literature.

In previous studies, EN and PN obtained from different plants were used as major phenolic compounds and antioxidant additives [11,45].

The highest total phenolic content was found to be 0.68 g PN/g (g punicalagin equivalent/g) in PPP. The other results obtained for PPP extract were an ABTS value of 25.34 mmol TE/g (mmol Trolox equivalent/g), DPPH value of 15.20 mmol TE/g and FRAP value of 4.75 mmol TE/g and a high level of antioxidant activity was observed. The measured equivalent (GTP and PPP) antioxidant levels are presented in Table 1.

There are different experimental studies carried out on the addition of GTP and PN extracts into meat and meat products [15,43]. The main objectives of these studies were to decrease the oxidative stress, to preserve food, to prolong the storage period and to prevent the possible pathogenic and chemical risks during the storage period. Added into meatballs for the same purpose in the present study, GTP, EP, PPP and PN showed antioxidant and antimicrobial activity and prevented the microbiologic and physicochemical risks.

The natural antioxidants and antimicrobial compounds added to meat products aim to replace the chemical preservatives. Thus, healthy meat products labeled “natural” would be more preferred [46].

**Figure 2:**
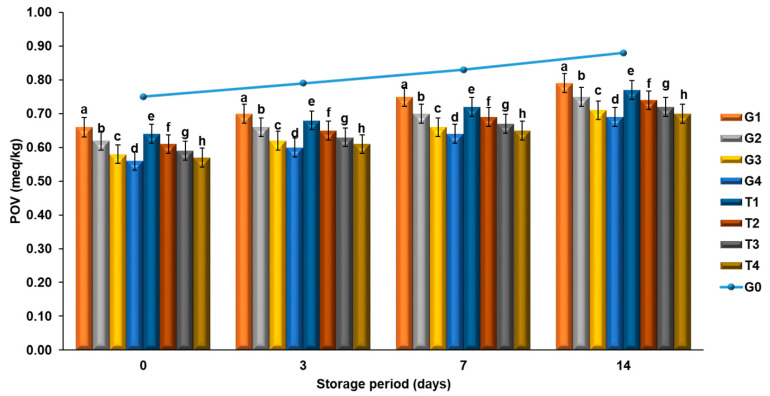
Changes in POV and meatballs during storage. The results are expressed as mean ± standard error. ^a–h^ For each day, columns representing different values are labeled with different letters.

In the previous studies, besides the antioxidant and antimicrobial activities, the addition of phenolic compounds into meat and meat products was also examined in terms of lipid oxidation levels [2,47]. The antioxidant and antimicrobial activity results obtained in the present study show similarities in terms of POV and TBARS and FFA values, such that the lowest level of increase in POV levels, that are most likely expected to increase during the storage period, was observed in the T4 group, having the highest antioxidant power (Figure 2).

As can be seen in Figure 2, the initial POV of meatballs in the control group was found to be 0.75 meqPOV/kg lipid. The POV showed a gradual increase in all the groups during the storage period (days 0, 3, 7 and 14) and that increase was found to be statistically significant in the control group, when compared to the other groups (*p* < 0.05). The highest and lowest POVs were observed in the G1 (0.5% GTP) and T4 (1% PN) groups, respectively.

Examining the POV findings from a general perspective, it can be stated that a lower level of increase was observed in the POVs of the groups to which GTP, EP, PPP and PN were added. As a result of this, it was observed that the peroxides spoiled more slowly and the progression of oxidation was delayed. The results of peroxide values were compared to those measured in meatballs enriched with rosemary. As stated by Georgantelis et al. [48], the increase in POVs in rosemary-enriched meatballs was found to be much slower.

The previous studies reported that the water-soluble and lipo-soluble natural phenolic antioxidants added into meatballs [46] could prevent oxidation during the storage period [49].

**Figure 3:**
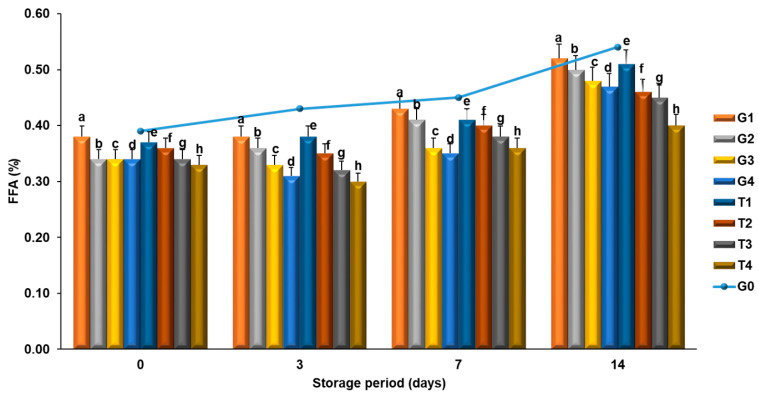
Effects of green tea powder, pomegranate peel powder, EP and PN on free fatty acids value of meatballs at different storage durations. The result was expressed as mean ± standard error. ^a–h^ For each day, columns representing different values are labeled with different letters.

Determining free fatty acids is a simple and applicable method for measuring the level of lipid hydrolysis. Knowing the FFA values of meat products during the storage period provides information about lipid and fat stability. The effects of extracts added into meatballs on the FFA levels can be seen in Figure 3. FFA values at the beginning of storage (0th day) were found to range between 0.39% and 0.33%. During the storage period, FFA values increased in the control group and, at the end of the storage period (14th day), the rate of increase in FFA values was found to be slow in the groups enriched with phenolic compounds (*p* < 0.05).

**Figure 4:**
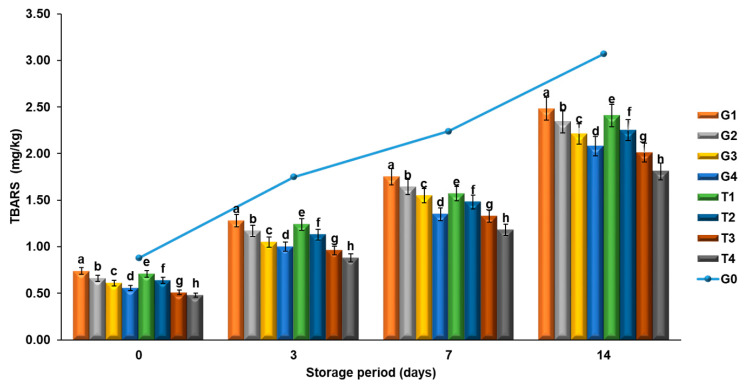
Changes in TBARS of meatballs during storage. The results are expressed as mean ± standard error. ^a–h^ For each day, columns representing different values are labeled with different letters.

A significant correlation was observed in the TBARS results of the groups enriched with GTP and PPP (R^2^: G1, G2, T1, T2 = 0.99; R^2^: G3, G4, T3 = 0.98; R^2^; T4 = 0.97). In the groups enriched with EP and PN, the TBARS values increased more slowly as the concentration increased (0.5−1%) (Figure 4). Phenolic compounds bind to metals by preventing the formation of free radicals [50]. The low percentage of phenolic compounds in the groups with increasing TBARS values was attributed to this reason. The decreasing values of lipid peroxidation inhibition might be due to the effects of high levels of bioactive compounds with antioxidant effects. In the groups enriched with GTP and PPP, high levels of EP and PN also contributed to the TBARS values. As emphasized by Ozer and Sarıcoban [51], TBARS values up to 1.59 mg MDA/kg have no negative effect on the consumer’s health. In the present study, this threshold value was not exceeded until the 3rd day of storage. Besides that, among the TBARS values measured on the 7th day of storage at +4 °C, there were also groups with values below the threshold value. Except for the G1 (1.75 mg MDA/kg) and G2 (1.64 mg MDA/kg) groups, the TBARS values of all other groups were found to be lower than 1.59 mg MDA/kg.

In a previous study, when compared to the control group, the TBARS increase in goat meat nuggets enriched with Moringa leaf extract during the storage period (45 days) was found to be slower. It was reported that the redox characteristics and bioactive content of Moringa extract might have caused the inhibition of peroxide [52].

In the present study, TBARS values on the 14th day of storage showed a decrease, when compared to the control group. When compared to the control group (control group = 100%), the T4 group decreased by 41.04% and the G1 group by 19.22%. In other words, in the T4 group, the conversion of secondary products occurring as a result of the decomposition of hydroperoxides into malonaldehyde was decreased. Fernandes et al. [46] reported that the decrease in TBARS values of thyme-enriched (*Origanum vulgare*) lamb burger samples suggests that the complex phenolic structure of herbal extract has an effect on the TBARS values and decelerates the conversion into malonaldehyde by preventing the formation of secondary products. In another study, Morsy et al. reported that lyophilized pomegranate peel nanoparticles were more effective in retarding lipid oxidation and improving the microbial quality and cooking characteristics of meatballs [53].

### 3.2. Determination of MIC and MBC

The minimum inhibition concentration can be defined as the “lowest microbial load” level inhibiting the highest among the microorganisms. In the present study, the inhibition levels were determined by using three different food pathogens (*E. coli*, *L. monocytogenes*, *S. typhimurium*) on the meatballs. The meatballs were enriched with different concentrations of green tea powder (GTP), pomegranate peel powder (PPP), EP and PN and they were set as groups. There were eight groups in addition to the control group. The groups were named G1 (0.5% GTP), G2 (1% GTP), G3 (0.5% EP), G4 (1% EP), T1 (0.5% PPP), T2 (1% PPP), T3 (0.5% PN) and T4 (1% PN). The studies in the literature were used for preparing the experimental setup. Having bioactive properties reported in many studies, GTP [22,54] and PPP [43,55] are the herbal extracts having natural antioxidant and antimicrobial activity.

The antimicrobial and antioxidant activities of natural extracts and standard phenolic compounds added into the meatballs at different concentrations were separately examined and it was found that the difference between the groups was statistically significant (*p* < 0.05). The results reported in previous studies corroborated that EP and PN, isolated from the herbal extracts, have antioxidant and antimicrobial activity [45,56].

MIC values of meatball samples were determined on 1st, 24th and 72th h. Different experimental setups were prepared for the meatballs enriched with pathogen microorganisms and GTP, PPP, EP and PN. The concentrations, at which the meatballs enriched with phytochemicals at different concentrations could inhibit the microorganisms (10^7^ CFU/mL) at minimum levels, were recorded.

**Figure 5:**
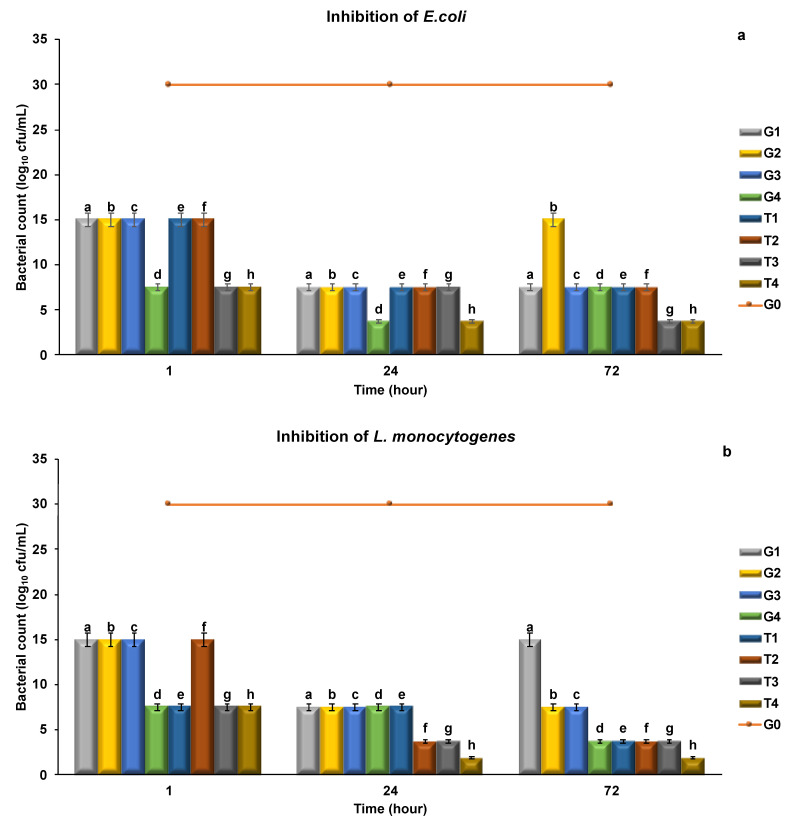

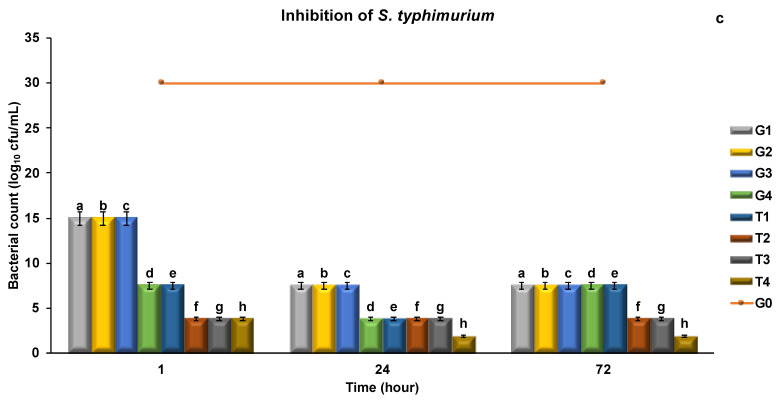
MIC values of meatballs determined at different times. (**a**) Inhibition of *E. coli*, (**b**) Inhibition of *L.monocytogenes*, (**c**) Inhibition of *S. typhimurium*. The results are expressed as mean ± standard error. ^a–h^ For each day, columns representing different values are labeled with different letters.

Among the pathogens, the highest MIC values measured against *E. coli* were observed at the end of 72th h. The results for GTP extract were 7.5, 15.0, 7.5 and 7.5 mg/mL, respectively (G1, G2, G3 and G4), and those for PPP extract were 7.5, 7.5, 3.75 and 3.75 mg/mL (T1, T2, T3 and T4) (Figure 5a).

Given the MIC results of *E. coli*, the highest results at the end of 24th h were recorded in the G4 and T4 groups (3.75 mg/mL). At the end of 72nd h, the same inhibition concentration (3.75 mg/mL) was achieved in the T4 group, but the level of inhibited microorganism load in the G4 group decreased (7.5 mg/mL). Examining the groups, it was determined that the T4 group could inhibit the pathogen until 72nd h, but the inhibition lasted 24 h in the G4 group. In the G2 group, however, the MIC value showed an increase at the end of 24th h (7.5−15 mg/mL).

Isolated from *Punica granatum L*. extract, punicalagin (2,3-hexahydroxyidiphenoilgallagil-d-glucose) is a main active tannin. While tannic acid can be hydrolyzed, catechin is included in the non-hydrolyzable group [57]. In the present study, the antimicrobial activity of PN was found to be stronger than that of EP. As reported by Mun et al., it was thought that the higher antimicrobial activity was because there were more water-soluble bioactive materials in the extraction solvent in PN-containing meatball groups [57].

Among the pathogens, the highest MIC values recorded for *L. monocytogenes* and *S. typhimurium* were found in the T4 group. GTP, PPP, EP and PN showed the highest level of antimicrobial activity against the pathogen *S. typhimurium*. As can be seen in Figure 5c, the MIC values recorded for *S. typhimurium* were lower than those recorded for other pathogens (*p* < 0.05).

Comparing the extracts in terms of MIC values, it was found that PPP > GTP. The standard was set to be PN > EP (Figure 5).

Damaging the bacterial cell membrane, inhibiting the fatty acid synthesis and preventing enzyme activity are among the antimicrobial characteristics of phenolic compounds. As a result of damaging the bacterial cell membrane, the toxin production capacity of bacteria decreases [58]. For this reason, among the pathogen bacteria selected in the present study, the metabolic activity of *S. typhimurium* was significantly damaged. *E. coli* showed a high level of resistance to the phenolic compounds and was subjected to the lowest level of inhibition.

In the literature, there are studies in which various herbal products (essential oil, herbal extract, etc.) were added into meatballs in order to benefit from antimicrobial properties [28,29,59].

In a previous study, the effects of essential oils (*Origanum vulgare*, *Thymus vulgaris*, *Rosmarinus officinalis*, *Cinnamomum zeylanicum* and *Salvia officinalis*) added into meatballs at different concentrations on the food-borne pathogens were tested. In conclusion, it was found that there was a statistically significant decrease in the microbial load of *L. monocytogenes* (0.125% *v*/*v*) [2]. In another study, it was reported that PPP extract showed antimicrobial activity on different pathogens, especially on *L. monocytogenes*, and it can be used as an alternative food preservative [43].

## 4. Conclusions

It was determined using the HPLC method that GTP and PPP extracts contain high levels of EP and PN. EP and PN standards were compared to the natural herbal extracts. It can be concluded that the herbal extracts (GTP and PPP) could successfully preserve the food-borne pathogens in raw meatballs stored at +4 °C for up to 72 h. The phytochemicals, which were found to have antioxidant properties, positively affected the chemical characteristics of the meatballs. The degradation of quality during storage was delayed for a minimum of 7 days. Thus, it can be seen that the selected phytochemical agents could be used in preserving the meatballs until cooked. The sensory and other quality parameters of GTP and PPP extracts, the phenolic composition and antimicrobial and antioxidant activities of which were determined, should be examined in more detailed research on this subject.

## Figures and Tables

**Table 1:** Antioxidant capacities (ABTS, DPPH and FRAP), total phenolic contents.

Antioxidants	GTP	PPP	GTP/EP	PPP/PN
DPPH (mmol TE/g)	11.44 ± 0.17	15.20 ± 0.63	1.10 ± 0.22	1.05 ± 0.27
FRAP (mmol TE/g)	4.91 ± 0.54	4.75 ± 0.27	0.76 ± 0.74	0.54 ± 0.38
ABTS (mmol TE/g)	19.07 ± 0.24	25.34 ± 0.38	0.94 ± 0.33	0.97 ± 0.21
Total phenolic content (g EP/g)	0.51 ± 0.12	-		
Total phenolic content (g PN/g)	-	0.68 ± 0.18		

Data represent mean values for each sample ± standard deviations (n = 3).

## Data Availability

The data are available by the corresponding author upon.

## Sample Availability

Samples of the compounds are available from the authors.
